# Outsider art in Croatia

**DOI:** 10.1017/S2045796024000349

**Published:** 2024-09-19

**Authors:** Daniela Bilopavlovic

**Affiliations:** Museum of Contemporary Art Zagreb, Zagreb, Croatia

Analysing and writing about the phenomenon of outsider creativity grows increasingly challenging with time, and the question arises whether this branch of art even needs a clear definition. Shouldn’t art inherently be self-sufficient, without the need for supplemental analysis and explication? Frequently, the suggestions and directives of art critics shatter the magic of personally experiencing and understanding an artwork. Nevertheless, the profession mandates categorization, for how can one discourse about an artist without pigeonholing them in a specific category? I will, therefore, attempt to single out and explain the concept of outsider art in Croatia and place it within the context of the recent art scene.

In the past, outsider art was often identified with naïve art, particularly in Croatia. While these two genres share similarities in their approach and attitude towards understanding art, their respective experiences diverge significantly. Naïve artists predominantly depict their external world, based on their experiences, whereas outsiders delve into their inner reality, often drawing inspiration from dreams and visions. Moreover, a distinction is made between recognizing the art of outsiders and art brutists, as highlighted by Nada Vrkljan Križić, the former founder and head of the Collection of Outsider Art at the Museum of Contemporary Art in Zagreb. In the foreword to the *Outsiders* exhibition catalogue, she references Hainy Widmers and Fritz Billeter, who say: ‘Here we are dealing with a sensitized artistic individual who, in their feelings, thoughts, actions, and life in general, breaks under the reality of the ruling majority and withdraws from the prevailing norms of behaviour and dominant worldview into their own conscious isolation, into a retreat into the subconscious.’ The creators of this fantastic world of art do not conform to social conventions in their works, they seek to penetrate the depths of the psyche and understand what constitutes the essence of human existence. For this reason, they have often been perceived as eccentrics and rarely placed on equal footing with academic artists. Numerous examples exist of outsider artists who, by fully surrendering to art, have transcended the confines of their mental boundaries, as well as the boundaries of art itself. Many among them only began to create freely after a mental breakdown.

Outsider Art in Croatia started to gain recognition in the 1990s, largely due to the efforts of Nada Vrkljan Križić (1940–2012), a curator who managed the Collection of Outsider Art at the Museum of Contemporary Art in Zagreb from 1997 to 2004. This period marked a pivotal shift in the perception of the art of the self-taught and catalysed changes in the exhibition policies on the contemporary art scene.

Thanks to Vrkljan Križić’s strong initiative, Croatian artists whom almost no one has ever heard of before emerged from obscurity. They were presented at two group exhibitions at the Museum of Contemporary Art: *Outsiders – Artists from the Other Side of the Mirror* (1998) and *Outsiders 2* (2000) at the exhibition space on Katarina Square in Zagreb. This was followed by the exhibition *Untamed* featuring the collections from the Musée de la Création Franche in Begles in 2004. Particularly significant was the exhibition of Karl Sirovy, first introduced to the public at a major solo exhibition and in the monographic catalogue *KARL SIROVY – Life and Work 1896-1948* at the Museum of Contemporary Art in Zagreb in the spring of 1993. His extensive and captivating body of work, which stands out in the Collection of Outsider Art, was discovered by Nada Vrkljan Križić. Following the curator’s retirement, the Collection of Outsider Art was dismantled and dispersed among other collections of the Museum of Contemporary Art.

It was re-established in 2016, and since then efforts have been made to collect works and expand the Collection, which now counts more than 600 works.

**Figure 1.**
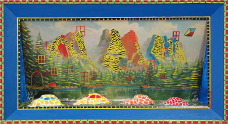
Gojislav Kalapač-Goja Daddy, buy me a car 2000 photo MSU archive.

**Figure 2.**
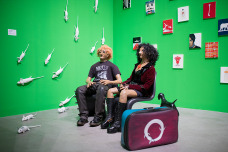
Margareta Vidmar and Exos Lucius Ido Travellers 2013–2020 photo Josip Bolonić.

Alongside Sirovy, the Collection features works by Croatian artists such as Krešimir Hlup, Drago Trumbetaš, Stjepan Bukovina, Emerik Feješ, Igor Lasić, Lovro Pavelić, Ljiljana Arar, Zvonko Zvone Bratić, Božidar Štef Golub, Gojislav Kalapač – Goja (Fig. 1), Drago Jurak, Dubravko Sertić, Margareta Vidmar (Fig. 2), Melita Kraus, Simon Petkovich, Ivan Švalj, Darko Brajković, Petar Brajković, works by Jasna Damnjanović and Goran Stojčetović from Serbia, and Katie Woznicki, an American with a Belgrade address.

At the new premises of the Museum of Contemporary Art, exhibitions of two ‘ladies from the margin’ were held: Margareta Vidmar’s *Jigsaw Puzzle* (2013) and Melita Kraus’s *The Mirror of Miraculous* (2017). Within the then-permanent exhibition of the *Collection in Motion*, an exhibition titled *Formula of the Senses – Works from the Collection of Outsider Art and Collections of the Museum of Contemporary Art* was opened in 2018, marking the inaugural presentation of the outsider art pieces.

These exhibitions raised significant public interest, which showed the need for further research and exhibition of outsider artists.

This was followed by a collaboration with the association Art Brut Serbia, which culminated in the exhibition *Dream Catchers* at the end of 2020. The exhibition included a cross-section of the contemporary art marginal – outsider and art brut scenes from Croatia and Serbia, marking the first such regional project and the beginning of collaboration. Twenty artists from Croatia and Serbia were presented, and their captivating, fantastical and imaginative works, created in various media, from drawings and collages to videos, transported us into their unique worlds. The exhibition *Dream Catchers* was also presented to the Serbian audience at the Museum of Naive and Marginal Art in Jagodina in November 2022.

In June 2022, the Museum of Contemporary Art hosted a group exhibition featuring marginal artists from the Austrian Infeld Collection, titled *Not a Dream, but an Experience – Outsiders from the Infeld Collection*. This exhibition featured a wide range of artists, members of the art brut/outsider scene from the first half of the 20th century to the present day, offering the audience a broader insight into the history, development and individual poetics of the artists. Works by 38 artists were presented, including major figures of art brut such as Adolf Wölfli, Jean Dubuffet, Gaston Chaissac, Scottie Wilson and Michel Nedjar, those created on the border between naïve art and revolt against petite bourgeoisie such as Ilija Bosilj, Sava Sekulić, Emerik Feješ, Dragutin Jurak and Louis Vivin, as well as members of the Vienna Actionists, Günter Brus and Otto Mühl.

It is extremely important to emphasize the importance of the existence and activities of outsider artists, especially within public cultural institutions – museums and galleries, as well as the existence of the Collection of Outsider Art within the Museum of Contemporary Art. Namely, the Museum of Contemporary Art in Zagreb stands as the sole institution in Croatia dedicated to collecting and exhibiting outsider artists. In this way, we draw attention to artists who create with different premises, unburdened by academic frameworks, maximally expressing their need for personal freedom. Although still insufficiently accepted in Croatia, this genre of art is slowly finding its place within the contemporary art scene and is becoming valued on par with ‘academic art’.

Daniela Bilopavlović Bedenik

Senior Curator, Museum of Contemporary Art Zagreb

Head of the Collection of Outsider Art
